# Barriers to and strategies for addressing the availability, accessibility, acceptability and quality of the sexual, reproductive, maternal, newborn and adolescent health workforce: addressing the post-2015 agenda

**DOI:** 10.1186/s12884-018-1686-4

**Published:** 2018-02-20

**Authors:** Caroline S. E. Homer, Sofia Castro Lopes, Andrea Nove, Michaela Michel-Schuldt, Frances McConville, Nester T. Moyo, Martha Bokosi, Petra ten Hoope-Bender

**Affiliations:** 1 Centre for Midwifery, Child and Family Health, University of Technology Sydney, City campus, Ultimo, NSW Australia; 2 Instituto de Cooperación Social Integrare, Calle Balmes, 30, 3° - 1, 08007 Barcelona, Spain; 3 UNFPA, 11-13 Chemin des Anémones, 1219 Chatelaine, Geneva, Switzerland; 4 WHO, Avenue Appia 20, 1211, 27 Geneva, Switzerland; 5 International Confederation of Midwives (ICM), Laan van Meerdervoort 70, 2517 AN The Hague, The Netherlands; 6 Novametrics Ltd, DE56 4HQ Duffield,Derbyshire, UK

**Keywords:** Health workforce, SRMNAH care, Availability, Accessibility, Acceptability, Quality, Effective coverage, Universal health coverage, Qualitative

## Abstract

**Background:**

In a post-2015 development agenda, achieving Universal Health Coverage (UHC) for women and newborns will require a fit-for-purpose and fit-to-practice sexual, reproductive, maternal, adolescent and newborn health (SRMNAH) workforce. The aim of this paper is to explore barriers, challenges and solutions to the availability, accessibility, acceptability and quality (AAAQ) of SRMNAH services and workforce.

**Methods:**

The *State of the World’s Midwifery* report 2014 used a broad definition of midwifery (“the health services and health workforce needed to support and care for women and newborns”) and provided information about a wide range of SRMNAH workers, including doctors, midwives, nurses and auxiliaries. As part of the data collection, 36 out of the 73 participating low- and middle-income countries conducted a one-day workshop, involving a range of different stakeholders. Participants were asked to discuss barriers to the AAAQ of SRMNAH workers, and to suggest strategies for overcoming the identified barriers. The workshop was facilitated using a discussion guide, and a rapporteur took detailed notes. A content analysis was undertaken using N-Vivo software and the AAAQ model as a framework.

**Results:**

Across the 36 countries, about 800 participants attended a workshop. The identified barriers to AAAQ of SRMNAH workers included: insufficient size of the workforce and inequity in its distribution, lack of transportation, user fees and out of pocket payments. In some countries, respondents felt that women mistrusted the workforce, and particularly midwives, due to cultural differences, or disrespectful behaviour towards service users. Quality of care was undermined by a lack of supplies/equipment and inadequate regulation. Against these, countries identified a set of solutions including adequate workforce planning supported by a fast and equitable deployment system, aligned with the principles of UHC. Acceptability and quality could be improved with the provision of respectful care as well as strategies to improve education and regulation.

**Conclusions:**

The number and scale of the barriers still needing to be addressed in these 36 countries was significant. Adequate planning and policies to support the development of the SRMNAH workforce and its equitable distribution are a priority. Enabling strategies need to be put in place to improve the status and recognition of midwives, whose role is often undervalued.

## Background

*The State of the World’s Midwifery 2014 (SoWMy2014): A Universal Pathway. A Woman’s Right to Health* [1] took its inspiration from the United Nations Secretary-General’s Every Woman Every Child initiative [2–4] to do everything possible to achieve the Millennium Development Goals (MDGs) by 2015 and work towards the development and adoption of a post-2015 agenda supportive of a continuing focus on maternal and newborn mortality and morbidity reduction.

The report was entitled *The State of the World’s Midwifery*, but it used a very broad definition of midwifery (“the health services and health workforce needed to support and care for women and newborns”) and therefore provided information about a wide range of sexual, reproductive, maternal, newborn and adolescent health (SRMNAH) workers, including doctors, midwives, nurses and auxiliaries. An effective SRMNAH workforce is critical to achieve universal health coverage (UHC) although it is likely that there are significant barriers in many countries to realising effective coverage of SRMNAH care [5].

For *SoWMy 2014*, 75 middle- and low-income countries, included in the ‘Countdown to 2015’ initiative [6], were invited to contribute to the analysis of SRMNAH needs. *SoWMy 2014* aimed to support policy dialogue between governments and their partners; accelerate progress on MDGs 4 and 5; identify developments in the three years since the *SoWMy 2011* report was published [7] and inform negotiations for and preparation of the post-2015 development agenda. In total 73 of the 75 Countdown countries participated in *SoWMy 2014*, the exceptions being Equatorial Guinea and the Philippines.

*SoWMy2014* was framed around the concept of ‘effective coverage’, with quality of care, equality and equity in reaching the most vulnerable members of society as the priorities. ‘Effective coverage’ includes the dimensions of availability, accessibility, acceptability and quality of services (AAAQ) included in the right to health [8] and the Tanahashi framework [9]. This provides not only an effective analytical approach, endorsed and adopted by United Nations agencies, but also a framework for analysis to inform country and global actions. The AAAQ framework is also useful as it addresses supply-side (availability and quality of care) and demand-side (accessibility and acceptability) factors.

Another aim of *SoWMy2014* was to be a means to catalyse action on SRMNAH services. Therefore, the study was seen as a way to collect rigorous data at country level, but also as an opportunity to encourage political buy-in for the willing contribution of often hard-to-find information, the political endorsement of best-available estimates where hard facts were not available, and a strengthening of policy dialogue on evidence, challenges, and solutions. Therefore, two main processes were used to collect data: 1) a self-completion questionnaire, which collected quantitative and qualitative data about education, regulation, professional associations, policy and planning frameworks and progress since *SoWMy2011*; and, 2) a one-day workshop involving national stakeholders and experts, to identify barriers and solutions for effective coverage of SRMNAH care. The workshops were considered as an important mechanism to engage a wide range of stakeholders and enable a participatory policy debate on the state of the country’s SRMNAH workforce. Based on the workshop data, the aim of this paper is to explore the barriers to, and strategies for, enabling SRMNAH services to be available, accessible, acceptable, and of quality.

## Methods

The development of *SoWMy 2014* was coordinated by the United Nations Population Fund (UNFPA), the World Health Organization (WHO) and the International Confederation of Midwives (ICM). Countries participating in *SoWMy 2014* were invited to hold a one-day interactive workshop, and 36 countries did so. The objectives of the workshop were to:

- Explain the conceptual framework and methodological approach behind *SoWMy 2014* and secure buy-in to the *SoWMy* research process.
- Elicit important qualitative information for the *SoWMy* analysis in relation to AAAQ, to enrich the information collected using the structured questionnaire (Table 1).
- Moderate a participatory policy debate on the state of the country’s SRMNAH workforce and strategies for strengthening it.

**Table 1:** AAAQ Framework

1.1. Availability
• Strategic intelligence on the health workforce
• Policy, regulatory and fiscal environments
• Education, training and professional support
• Financing supply
• Bilateral, multilateral and regional partnerships
1.2. Accessibility
• Geographical, temporal and financial barriers to access
• Stewardship, management and equitable deployment
• Referral across health services
• Equitable access for vulnerable groups
• Retaining health workers
1.3. Acceptability
• Increasing population demand for services
• Workforce skill-mix, competencies, socio-cultural needs
• Responsiveness to population-specific needs
• Oversight and accountability
1.4. Quality
• Patients’ interests
• Standards, accreditation, regulation
• Linking professional, community and consumer organizations
• Managing patient risk
• Workforce management, performance and monitoring systems

UNFPA and WHO focal points in each country were invited to arrange the workshop. In each country, a list of about 25 participants was drawn up in consultation with the Ministry of Health. Those invited to the workshop included a wide range of stakeholders such as: representatives from the Ministry of Health, advisers, advocates, health service managers and leaders, SRMNAH workers, professional associations (including midwifery, obstetrics and gynaecology, nursing), education providers, regulators, international and donor organisations, non-governmental organisations, the media, the private sector and academia. Across the 36 countries (Table 2) there were about 800 participants (between 6 and 54 per workshop).

**Table 2:** SoWMy2014 countries (*n* = 36) who held a workshop, by WHO region

Africa (*n* = 20)	South-east Asia (*n* = 5)	Western Pacific (*n* = 1)	Americas (*n* = 3)	Eastern Mediterranean (*n* = 5)	Europe (*n* = 2)
Benin, Burkina Faso, Chad, Congo, Democratic Republic of Congo, Côte d’Ivoire, Ethiopia, Guinea, Liberia, Malawi, Mali, Mauritania, Mozambique, Niger, Nigeria, Rwanda, Sierra Leone, Tanzania, Togo, Zambia	Bangladesh, India, Indonesia, Myanmar, Nepal	Lao People’s Democratic Republic	Brazil, Haiti, Mexico	Afghanistan, Morocco, Pakistan, Somalia, South Sudan	Kyrgyzstan, Tajikistan

Prior to the workshops, potential participants were informed about the procedures involved, the optional nature of the process and the possibility to withdraw their consent at any point. To encourage open and honest discussion, the workshops were held under ‘Chatham House Rules’ [10] and not video or audio recorded.

Moderators facilitated the workshops using materials provided by the SoWMy secretariat. Countries were asked to address AAAQ of the SRMNAH care system in the workshops, and a series of guiding questions were used. A rapporteur attended the workshop, and took detailed notes of what was said. A written report of each workshop was produced using a template. The draft report was sent to the workshop participants so that they could check that it was an accurate record. Once they had checked the draft, the report was submitted to the SoWMy secretariat via an on-line submission system.

Data from the workshops undertaken in French, Portuguese and Spanish were translated into English. The data were then imported into a qualitative software program (N-Vivo) and a content analysis approach was used. Qualitative content analysis is an approach that goes beyond counting words or responses to interpretation and classification in categories that represent similar meaning [11]. It is a recognised method for the subjective interpretation of the content of textual data through the systematic classification process of coding and identifying themes or patterns [11–14]. It is also a recognised method of analysing documents or written responses [15] which is particularly applicable for these workshop reports.

A content template analysis approach [16] was used initially to explore the barriers using the AAAQ [8, 9] and ICM’s pillars of Education, Regulation and Association [17]. The AAAQ (availability, accessibility, acceptability, quality) framework (see Table 1) was used as a template to develop the initial codings [16]. This process of coding [15] ensured that the AAAQ framework was the basis for the analysis of these questions which is in keeping with the overall principles underpinning *SoWMy2014*.

## Results

### Barriers to enabling AAAQ

#### Availability

The first dimension of effective coverage is availability, i.e. are there enough SRMNAH workers to meet the SRMNAH needs of the population? Almost all countries made a comment about the availability of care being limited by insufficient numbers of SRMNAH workers in the workforce. This was partly due to a perception of insufficient numbers being educated, but in many countries the efficient and timely deployment of staff after the completion of training was highlighted as a problem. This included a lack of planning and coordination between production and deployment of SRMNAH workers, long waiting times for posting following graduation resulting in trained staff being unemployed with subsequent loss of skills and confidence or loss due to external migration. Some countries also felt that SRMNAH workers moving into the private sector meant they were lost to public sector services. Three countries specifically reported SRMNAH workers refusing to report to their duty station for political or personal reasons and because of fears of personal security. In addition, more than a third of countries reported that there were “*delays in the deployment”* to areas of need and SRMNAH workers were sometimes “*relocated to the workplace where their ... skills may not be used to the fullest”* (e.g. provincial/district health office/financial office).

One consequence of lack of overall availability is that the available health workers tend to choose to work in locations that they perceive as more desirable, leaving few or none to work in other locations. In all 36 workshops, comments were made about geographic maldistribution of the available workforce, for example: “*workers do not choose to work in rural areas as there are fewer opportunities for their families due to inadequate working conditions and an inappropriate distribution of resources among provinces and regions*”. A lack of housing for health workers in both rural and urban settings, an uneven distribution of health training institutions, no incentives to go to rural areas and a lack of a human resources for health (HRH) plan or strategy were all highlighted as significant barriers to accessibility. Insufficient numbers of health professionals were educated and they were often disproportionately located in urban areas “*Most of the population is in the rural areas but most of the workforce is in the urban areas*” was a common comment in this section.

#### Accessibility

The second dimension of effective coverage is accessibility: even if there are enough SRMNAH workers, women and newborns may not be able to access their services, e.g. due to being unable to make the journey to a health facility or being unable to afford to pay for care. The lack of an integrated approach to addressing accessibility, and subsequent availability, was a strong theme with a lack of vision or national planning hampering efforts at all levels.

Specific issues related to geographic accessibility were:

- Physical barriers and challenges – especially limited access to transport to and from services due to cost, availability and problems with roads, safety and security and the impact of natural disasters.
- Poor communication networks hindered effective referral and advice-seeking.
- Inadequate places for women to wait near a health facility when towards the end of pregnancy and awaiting labour.
- Lack of integration or referral between primary health care and higher levels.

Issues relating to financial accessibility/affordability included:

- High cost of transportation for care or referral.
- Cost of health services and medicines, especially in rural areas.
- Informal payments on all levels of care, including to access medicine and commodities to buy essential items such as surgical gloves.

Problems with financial accessibility were further compounded by “*a lack of community understanding that access to free or subsidised care was available*” and thus the “*demand was low*”. The low status of women also meant that they had limited influence over decisions on spending household funds for SRMNAH care and sometimes were not deemed sufficiently “*valuable*” to require access to funds for care. A lack of “*access to health insurance schemes for maternal and newborn health*” care meant many women could not access care.

Interestingly, in some countries where financial accessibility had improved, the health system was unable to meet the new demand. For example, one country reported that “*health services were not equipped adequately to meet the increasing [demand]*”.

#### Acceptability

The third dimension of effective coverage is acceptability: even if SRMNAH workers are available and accessible, if the care they provide is not acceptable to women and their families, people may choose not to use their services. Acceptable care requires that service providers should be respectful of medical ethics and culturally appropriate. The acceptability of care was perceived to be limited by “*discrimination based on gender, culture or religion*”, the availability of a culturally appropriate and skilled workforce, whether due to a shortage of women in the SRMNAH workforce or the “*lack of trust in the health workforce*” in terms of their skills and competencies. As noted in the next section on quality, a lack of clarity about the role of a midwife meant that at community and institutional level, midwives were not always accepted as a quality provider of SRMNAH services and were thus invisible to the community. The workshop participants perceived that communication barriers and the age of the care provider (younger women especially were not seen as acceptable options) limited acceptability of care.

Significantly, almost all countries recognised that inhumane and undignified care was a serious impediment to acceptability of care. This included a “*harsh, unfriendly and judgemental attitude from health workers*”, “*a lack of time and privacy*”, “*aggressive, offensive behaviour or disrespect from health workers*” and health facility protocols which did not permit women to have support in labour from a partner, family member or friend. These attitudes and behaviours meant that many women choose not to seek care and/or to give birth unattended.

#### Quality

The fourth dimension of effective coverage is quality. Even if the SRMNAH workforce is available, accessible and acceptable, poor-quality care can make it ineffective. Quality of care was felt to be limited by a “*lack of essential supplies in health facilities*”. Almost half the countries highlighted the lack of basic equipment and essential drugs such as oxytocin and magnesium sulphate, and many also mentioned the high number of unskilled staff, the limited number of facilities able to provide emergency obstetric care, the inconsistent opening hours of facilities and the lack of reliable government funds to provide services. These issues were particularly prevalent in rural areas where supplies were uneven and inconsistent in the same way as the health workforce (see ‘accessibility’ above).

A lack of clarity about the roles of certain cadres of SRMNAH worker (most notably midwives) and therefore about their ability to provide skilled and competent care meant that the SRMNAH workforce was not always able to undertake tasks even when educated to do so (including not being authorised to prescribe emergency/life-saving medications) and were therefore considered an underutilised human resource. Frequent rotations and de-skilling of midwives meant that “*midwifery skills were lost as staff covered paediatrics, critical care units and medical areas*”. A lack of basic education for midwives and “*poor career pathways or continuing professional development opportunities*” were perceived to affect midwives’ morale which in turn affected their motivation to provide high quality services.

The lack of regulation and licensing for the health workforce was acknowledged by more than a third of participating countries. This includes the lack of clarity from the community about the roles of SRMNAH workers (especially midwives) or even the need for regulation, no linkage of continuing professional development with annual renewal of licences, a lack of national standards and limited supervision and monitoring. These are all perceived to contribute to decreased quality of care.

### Strategies and solutions to enable AAAQ

Having identified the barriers and challenges to AAAQ, workshop participants were asked to put forward their ideas for addressing these barriers and challenges.

#### Availability

The importance of HRH planning was a key solution identified by all 36 countries. This included having an HRH strategy and human resources management skills, ensuring budget allocations were applied for SRMNAH posts, mapping the workforce, simplifying and standardising the terminology used to define health workers, job descriptions and roles and applying national or international norms and standards in terms of health worker to population ratios.

Countries recognised that HRH planning should include streamlined and harmonised recruitment and deployment procedures to ensure timely deployment; skilling up of all cadres especially at sub-national level; and particular attention to HRH policy and strategy in rural areas. The need for incentives to work in rural areas was highlighted by nearly half the countries including “*support for girls and young women in rural communities to receive basic education before training as [SRMNAH workers]*” and “*security for staff*”. Other suggested strategies included “*imposing 2 years of rural service for all*”, “*improving infrastructure in the rural areas*” and “*accommodation and schooling for kids*”.

#### Accessibility

The need for a national policy to ensure UHC was recognised by most countries as being critical to addressing financial accessibility. Strategies included “*systems of health insurance*”, “*streamlined voucher or payment systems*” and the “*removal of informal payments or out-of-pocket expenses*”. Affordable, safe transport for women to get to and return from services was recognised as essential for geographic accessibility.

#### Acceptability

Acceptability solutions focused on the provision of respectful care. Language skills, cultural sensitivity, respect for privacy, adequate time to spend with women and reduced waiting time were all identified as important. Clean health facilities were seen as important as this also helped women feel respected and cared for. Education for women, both to enable them to know when to seek care and as a means to have a future health workforce, community awareness of the importance of SRMNAH care, advocacy for a human rights-based approach and the involvement of men in SRMNAH issues were other strategies to address acceptability recorded by almost one third of countries.

The “*inclusion of respectful care in curricula*” and “*continuing professional development*” for all SRMNAH workers was seen as essential. This may also build self-confidence and enhance the self-esteem of staff, which in turn is likely to result in more acceptable care being provided. Mentoring, the use of role models in education and practice and supportive supervision by regulatory bodies, professional associations and employers was also needed to facilitate respectful care to be practised. A small number of countries called for punitive measures to address malpractices and misbehaviours.

#### Quality

Many of the strategies and solutions to address acceptability will also address quality. Quality is also addressed through effective education, regulation, deployment, “*clarity about job descriptions and roles and responsibilities*” and appropriate “*salaries and other incentives*”. In-service training and support in teamwork and “*task sharing or shifting with appropriate supervision*” were also seen as useful approaches.

Box 1 Summary of the barriers and strategies or solutions to enable AAAQ

**Table:** 

Barriers	Strategies and solutions
*Availability* • Lack of efficient and timely deployment of staff • Geographic maldistribution of the workforce	*Availability* • Human resources for health strategic planning and human resources management • Streamlined recruitment and deployment procedures • Incentives to work in rural areas
*Accessibility* *Geographic accessibility* • Limited access to transport • Problems with roads, safety and security • Poor communication networks • Lack of integration or referral between primary health care and higher levels • Impact of natural disasters *Financial accessibility/affordability* • High cost of transportation for care or referral • Cost of health services and medicines, • Informal payments to access medicine and commodities	*Accessibility* • Affordable, safe transport for women to get to and return from services • Improved referral and integration • National policy to ensure universal health coverage including health insurance systems
*Acceptability* • Care is not respectful and/or culturally appropriate • A lack of clarity about the role of a midwife as an acceptable care provider	*Acceptability* • Provision of respectful care – attitudinal change • Inclusion of respectful care in curricula and continuing professional development • Education for women and the community about expectations of respectful care and the role of midwives • Involvement of men
*Quality* • Lack of essential supplies • Limited number of facilities able to provide emergency obstetric care • Lack of up to date pre-service and in-service education • Lack of clarity about the roles of certain cadres of SRMNAH worker • Lack of regulation and licensing for the health workforce	*Quality* • Up to date and accredited pre-service education programs and teachers • Ensuring adequate clinical experience and practice • Attracting quality students • Essential supplies and commodities available when needed • In-service training and support • Clarity about job descriptions and roles and responsibilities • Regulation, national recognition, standards, licensing and supervision and monitoring • Support for professional associations

### Using education, regulation and association as enablers of AAAQ

The ICM pillars of education, regulation and association [17] were examined as a framework to achieve AAAQ. The education pillar had three main streams – 1) improving the faculty and facilities; 2) attracting quality students through the recognition and value of the role of the SRMNAH worker; and 3) the importance of clinical experience and practice. In terms of regulation, the need for national recognition, standards, licensing and supervision and monitoring were the main areas of need. Professional associations were highlighted as a mechanism to provide leadership and advocacy, influence and inform policy and enable capacity building (Table 3).

**Table 3:** A summary of the strategies by ICM pillars of education, regulation and association

Education
• Improving quality of educational institutions including better facilities, learning environments (including e-learning) and renewed and updated curricula
• Increasing the number of training facilities in rural areas • Accreditation and regulation of education providers to ensure quality
• Faculty development programs for educators and incentive plans to attract and retain quality educators
• Continuing professional development opportunities
• Skilled preceptors/clinical instructors and appropriate practicum sites to increase and re-vitalise practical, hands-on training in hospitals
Regulation
• An understanding of the importance of the role of the SRMNAH worker and the need for regulation at government and community levels and expressed in national policy
• A Council or Board to lead regulation
• National standards on which to guide course accreditation and licensing
Association
• Leadership and advocacy
• Collaborate with government and regulators
• Influence national and sub-national policy and planning
• Encourage health workers to be members – build capacity and provide mentorship

## Discussion

Workshops were an effective way to gather cross-national information on the barriers to effective coverage of SRMNAH care. Many of the issues identified are in line with other research which highlights the challenges to quality SRMNAH care and the need to take a systematic and step-wise approach to health system strengthening [18]. Interestingly, even though these data were provided predominately by health care professionals, policy makers from governments and professional associations, the findings support research from the perspectives of women about SRMNAH care [19].

It is clear that some of the proposed solutions or enablers of AAAQ require differing levels of investment and effort at a range of levels. Some require attitudinal change in health workers (ensuring respectful care; involvement in professional associations), others require a reallocation or re-prioritisation of resources (HRH planning processes, incentives to work in rural areas) while others will require significant investment (curriculum development, housing in rural areas, health insurance systems). These will vary from country to country and will need identification according to need and feasibility.

The workshops highlighted issues in the supply side (availability and quality of care) in particular, as well as challenges on the demand side, e.g. in accessing the available workforce. Participants expressed concerns about deficiencies in the size and quality of the SRMNAH workforce, the rural – urban divide in terms of services and providers, and the need to recognise and utilise all SRMNAH workers to their full capacity. Change will be difficult to fully implement for all countries regardless of the solution being evident. Political will and commitment that is backed up by resources, legislative and regulatory reform and community support is required at global, national and regional levels. In line with this, the International Labour Organisation has identified the need to monitor gaps and deficits in legal coverage, availability and affordability of services as well as assess the financial deficit that needs to be closed [20]. This will also include an assessment of the access barriers such as fragmentation of coverage. It is clearly the responsibility of governments to ensure that there is infrastructure to support an effective health workforce including housing, education, security, transport and utilities in rural areas so that there will be, in the long run, no area which is ‘hard to reach‘. It is recognised that improving living conditions for health workers and their families with investments in infrastructure and services has a significant influence on a health worker’s decision to locate to and remain in rural areas [21].

One of the potential solutions is a reconsideration of the way services are delivered and the way care is arranged. The provision of first level SRMNAH care as close as possible to women’s homes and communities (while ensuring access to consultation and referral transportation to higher-level services) will address accessibility issues while effectively utilising the health workforce. In some contexts, it may be necessary to upgrade specific facilities (e.g. well-functioning facilities with sufficient staff) or to incentivise those facilities in order to achieve an equitable geographic distribution of services [22]. Efficient use of health workers and collaboration with community-level lay workers and volunteers will facilitate access to cost-effective care, especially for women and families in geographically remote or urban poor settings without transportation. If referral mechanisms are available and adequately functioning, quality midwife-led care can be delivered at community level, reducing unnecessary delays and improving health outcomes [5]. First-level midwife-led units [23–27] could be established within reach of communities, supported by community health workers and traditional birth attendants who assist women to access the health system and facilitate respectful, culturally sensitive care [28].

Many health workers globally work in difficult, unsafe, isolated and poorly equipped settings and themselves experience gender-based violence, poor salaries and working conditions and a lack of access to continuing professional development; all of which impede their ability and/or motivation to provide high-quality care [29, 30]. Poor working conditions undermine their ability or willingness to continue practising: many choose to leave the workforce due to frustration with their position and role [30] or because they reach an arbitrary retirement age. A recent survey of 2470 midwifery personnel who provided care to childbearing women and their newborns in 93 countries showed that while midwives were committed to providing the best quality of care for women, newborns and their families, they were frustrated by the realities they experienced that constrain their efforts [30]. To further highlight this, a systematic review has also shown that there are significant social and cultural, economic and professional barriers that prevent the provision of quality midwifery care in low and middle income countries and these need to be addressed to bring about improvements in the quality of care [31].

Some of the strategies to bring about improvements in the quality of care include having an enabling professional environment that will support effective education, regulation and professional association [17]. This means that SRMNAH workers can develop meaningful relationships with women, with occupational autonomy and flexibility, so that they control, organize and prioritise their own work; have access to supportive supervision, reflect on practice with peers and colleagues, share ideas and information and optimise service provision [29, 32, 33]. This is more likely to result in the provision of quality care (supply-side) and address demand side factors especially related to acceptability of care from the perspective of women.

It was evident that high-quality education, continuing professional development and career pathways are critical to addressing many of the AAAQ challenges especially in relation to the provision of quality care which is much needed to promote accessibility and ensure acceptability [34]. This includes: making a career as an SRMNAH worker attractive; providing educational pathways with sufficient opportunities for clinical experience; having well-prepared faculty and appropriately resourced programmes; developing or applying accreditation systems with measurable standards and criteria; providing a safe and conducive learning environment; and facilitating community engagement to ensure that what health workers are taught meets community needs and incorporates respectful care and socio-cultural sensitivity [35]. Quality of initial education and ongoing training and support must ensure that SRMNAH workers remain competent to do their job effectively, can gain advanced clinical skills if desired or follow leadership and management training to become SRMNAH leaders. Continuous professional development programmes will increasingly be delivered through information and communications technology using blended learning that includes eLearning and face-to-face time, potentially in education hubs, either locally or regionally. A strong and functional regulatory system is also necessary with registration and licensing, incorporation of internationally consistent standards and codes, the accreditation of education programmes and continuing professional development frameworks so that periodic re-licensing and evidence of continued competence can be monitored. Vibrant and committed professional associations can provide: a point of leadership and advocacy, lobbying for improved working conditions (including flexible hours, adequate remuneration, leave, housing, transport, safety and security); opportunities for career development, promotion and incentives for retention; access to information and evidence for enhancing practice through continuing education and research. Effective support may include twinning models between individuals or associations [36]. Development, training and support are required to assist the sustainability of associations and to enable members to work at political and government levels and exercise advocacy both for women generally and for SRMNAH workers.

An enabling practice environment includes access to effective and reliable consultation and referral networks [37] as well human resources development, management and capacity building. The workshops highlighted the need for every country to have a minimum dataset on their SRMNAH workforce to enable efficient workforce planning and determination of the appropriate SRMNAH team [38]. As result, *SoWMy2014* [1] suggests that each country should routinely collect a minimum dataset of 10 data items to enable efficient planning of the SRMNAH workforce. This minimum dataset includes: headcounts of the professions involved, percentage of time spent on SRMNAH, roles, age distribution, retirement age, length of education, enrolments into, attrition and graduation from education, and voluntary attrition from the workforce [1]. Innovative technology can help build projections and future workforce scenarios from anticipated changes and challenges to support policy decisions and fit-for-purpose adjustments more accurately [38]. Undertaking a comprehensive SRMNAH workforce assessment would also address the future workforce needs and assist countries to determine how to best deploy their workforce to deliver essential SRMNAH interventions at scale and quality with universal access [38].

Performance review and development is an important component of human resources management. This will identify the needs of individuals and services, including learning needs to maintain competence, the successes and challenges of their work and allows service delivery to meet the needs and culture of the local population. Performance review and development will identify the need for continuing professional education and quality improvement. Advancing along a career path is an important component of job satisfaction. A career matrix can enable people to undertake a range of roles at different times in their career while ensuring that knowledge and skills remain in the health-care system and the professions. Developing opportunities for staff to move into other roles including extended clinical roles, education, management or research will require formal development options including faculty development programmes. New technologies [39] can enable ‘virtual’ schools or e-learning programmes to be established and widely accessed.

Inter-professional collaboration in education and practice is likely to ensure a fit-for-purpose workforce is developed [40]. Implementing inter-disciplinary teamwork and collaboration involves: learning together to create a ‘collaboration-ready’ workforce, and; respecting and building on each other’s disciplines and competences, communicating with one another and handing over to ensure continuity and consistency of care for women, and debriefing together to learn from errors.

The data from the workshops were an important contribution in the development of a pathway for policy and planning that is known as *Midwifery2030* [1, 41]. *Midwifery2030* is based on what women, adolescents and newborns need and want from an effective health system, it presents a coherent policy and planning vision to guide the provision of services across the two continuums of SRMNAH care: from pre-pregnancy to post-partum/postnatal and from communities to referral hospitals. *Midwifery2030* focuses on addressing supply and demand side issues through increasing the AAAQ of health services and health providers, reaching a greater proportion of the population (increasing coverage) and extending the basic and essential health package (increasing services) while protecting against financial hardship (increasing financial protection).

These workshops were conducted prior to the end of the Millennium Development Goals era and before the Sustainable Development Goals (SDGs) were instituted. Many of the SDGs clearly impact on SRMNAH and the ability of the SRMNAH workforce to provide quality care, including the goals that involve gender equity, education, poverty, hunger and water and sanitation [42]. The SDGs highlight that a holistic, equitable and coherent outlook is needed and recognises that there are multiple dimensions, inter-linkages; cross-cutting issues and partnerships that need to be addressed. Addressing these broader issues will impact on SDG 3 and ultimately the capacity and capabilities of the SRMNAH workforce in the SDG era (2015–2030).

### Limitations

Utilising workshop data in this way has limitations that need consideration. Only half of the *SoMWy 2014* countries facilitated workshops and hence they may not be reflective of all 73 countries in the report. It is not clear why some countries chose not to facilitate workshops. The countries that did facilitate workshops however represent all the global regions and many of the findings are in line with knowledge from other countries. Most of the participating countries were in Africa, which reflects the fact that most of the Countdown to 2015 countries were in this region. However, it is possible that some issues are region-specific and thus not generalisable to other parts of the world. Despite these limitations, the findings are likely to be broadly representative of many low- to middle-income countries facing challenges providing quality, universal SRMNAH care at scale.

The wide variation in the number of participants per workshop may have affected the focus and direction of the discussions, and therefore the comparability of the workshops from the different countries. In addition, the small number of participants in some countries may be viewed as a limitation, although we cannot identify the exact impact this may have had on the findings. However, the fact that the data emanating from the workshops are consistent with existing literature indicates that they are robust.

The translation of the workshop reports into English may have introduced some bias into the data analysis. The risk of this was minimised by the use of professional translators to maximise comparability and consistency of terminology, and there were no obvious inconsistencies between countries providing data in English and those providing data in other languages.

## Conclusions

This study allowed the identification of barriers to and possible solutions for improving the AAAQ of SRMNAH services in low-resource settings. It brought further attention to well-known and persistent demand and supply side bottlenecks that exist in health systems in these settings, showing that the number and scale of barriers still needing to be addressed in these 36 countries is significant. Particularly, it provided important insight on how these specifically affect AAAQ, and how addressing them could contribute to the achievement of UHC by 2030. Adequate planning and policies that support the development of the SRMNAH workforce and its equitable distribution, as well as the recognition of all SRMNAH professionals as a valuable part of the wider health workforce, is a priority particularly to ensure that demand-side factors such as acceptability to women are addressed. The role of the midwife is often undervalued despite the evidence supporting their actual and potential contribution to better SRMNAH outcomes. An enabling environment based on education, continuous professional development, regulation and licensing could enhance midwives’ role and improve their ability to provide quality of care towards UHC.

## Abbreviations

AAAQ: availability accessibility acceptability quality
HRH: human resources for health
ICM: International Confederation of Midwives
MDG: Millennium Development Goal
SDG: Sustainable Development Goal
SoWMy: State of the World’s Midwifery
SRMNAH: sexual, reproductive, maternal, newborn and adolescent health
UHC: universal health coverage
UNFPA: United Nations Population Fund
WHO: World Health Organization
